# Serological Evidence of Akabane, Bluetongue, and Bovine Ephemeral Fever Virus Exposure in Feral Water Buffaloes from Northern Australia

**DOI:** 10.3390/v18030363

**Published:** 2026-03-16

**Authors:** Andrew M. Adamu, Andrew J. Hoskins, Cadhla Firth, Bruce Gummow, Roslyn I. Hickson, Paul F. Horwood

**Affiliations:** 1 Australian Institute of Tropical Health and Medicine, James Cook University, Townsville, QLD 4811, Australia; andrew.adamu@my.jcu.edu.au (A.M.A.); roslyn.hickson@csiro.au (R.I.H.); 2 Centre for Tropical Biosecurity, James Cook University, Townsville, QLD 4811, Australia; bruce.gummow@jcu.edu.au; 3 Commonwealth Scientific and Industrial Research Organisation, Townsville, QLD 4811, Australia; andrew.hoskins@nailsma.org.au; 4 College of Science and Engineering, James Cook University, Townsville, QLD 4811, Australia; 5 Northern Australia Indigenous Land and Sea Management Alliance, Darwin, NT 0810, Australia; 6 School of Environment, University of Queensland, Brisbane, QLD 4072, Australia; 7 College of Science and Engineering, James Cook University, Cairns, QLD 4878, Australia; cadhla.firth@gmail.com; 8 Department of Production Animal Studies, Faculty of Veterinary Science, University of Pretoria, Pretoria 0002, South Africa; 9 Australian Institute of Tropical Health and Medicine, James Cook University, Cairns, QLD 4878, Australia; 10 Centre for Tropical Biosecurity, James Cook University, Cairns, QLD 4878, Australia

**Keywords:** buffaloes, Akabane virus, bluetongue virus, bovine ephemeral fever virus, Australia, dried blood spots

## Abstract

Water buffaloes in northern Australia occupy tropical wetlands where conditions favour the proliferation of arthropod vectors and the transmission of vector-borne livestock diseases. However, their role in maintaining economically important arboviruses such as Akabane virus (AKAV), bluetongue virus (BTV), and bovine ephemeral fever virus (BEFV) remains poorly understood. These three viruses cause significant production losses in cattle and pose ongoing surveillance challenges in remote areas. To assess exposure to these viruses, a convenience sample of feral water buffaloes from the Northern Territory, Australia, was collected. Commercial enzyme-linked immunosorbent assays (ELISAs) were used to detect antibodies against AKAV, BTV, and BEFV in 119 samples stored as dried blood on filter paper. Seroprevalence was 18.5% for AKAV, 66.4% for BTV, and 15.1% for BEFV. These results are consistent with previous serological studies in northern Australian cattle, confirming the circulation of these pathogens in the region. Our findings demonstrate that water buffaloes are exposed to these economically important arboviruses and may contribute to their maintenance, highlighting the need to consider feral buffalo populations in regional arbovirus surveillance strategies and livestock disease management.

## 1. Introduction

Water buffaloes (*Bubalus bubalis*) are native to Asia and were introduced into northern Australia for meat production between 1825 and 1843 [1]. In the absence of natural predators and with abundant suitable habitat, feral buffalo populations expanded dramatically across tropical northern Australia, particularly in floodplains and wetlands. This expansion has resulted in substantial environmental impacts, including habitat degradation, altered hydrology, and impacts on native flora and fauna [1,2]. Feral buffaloes frequently share habitat with cattle in northern Australia, raising concerns about disease transmission between these species. Such transmission could have significant implications for livestock health and productivity, as well as for disease control programmes [3]. For example, the Australian government’s Brucellosis and Tuberculosis Eradication Campaign (BTEC) in the 1980s targeted both cattle and feral buffaloes as part of a successful eradication effort, resulting in dramatic reductions in buffalo populations in some areas; for example, in Kakadu National Park, buffalo densities declined from 5.6 to 1.2 animals km^−2^ [4].

Three arthropod-borne viruses (arboviruses), Akabane virus (AKAV), bluetongue virus (BTV), and bovine ephemeral fever virus (BEFV), are the major vector-borne viral pathogens affecting domestic and wild ruminants in Australia. BTV belongs to the genus *Orbivirus* and family *Sedoreoviridae* and causes bluetongue disease, an economically important, non-contagious viral disease of ruminants [5]. Clinical disease is most severe in sheep, white-tailed deer, and pronghorn antelope, while cattle, goats, and camelids typically experience subclinical infections but can serve as reservoir hosts [6]. Bluetongue is listed as a notifiable disease by the World Organisation for Animal Health (WOAH) due to its severe economic impact through morbidity, mortality, and trade restrictions [7,8]. AKAV is classified in the genus *Orthobunyavirus* (order Bunyavirales, family *Peribunyaviridae*) within the Simbu serogroup. It has been reported across many countries in Africa, Asia, the Middle East, and Australia, infecting both wild and domestic ruminants [9]. The virus is known to cross the placenta, resulting in abortion, stillbirth, and congenital abnormalities (arthrogryposis-hydranencephaly syndrome) primarily in cattle and sheep [10]. BEFV is classified in the genus *Ephemerovirus*, family *Rhabdoviridae*, and causes bovine ephemeral fever, commonly known as ‘three-day sickness’, a potentially debilitating but self-limiting febrile illness in cattle and water buffaloes [11]. While mortality is typically low, the disease can cause significant short-term production losses, particularly in milk yield and body condition [12].

Although serological evidence confirms the circulation of these three arboviruses in cattle across northern Australia [13,14], the role of feral water buffaloes as potential reservoirs or amplifying hosts has not been investigated. Given their abundance in vector-rich wetland habitats and potential contact with cattle, understanding arbovirus exposure in buffalo populations is important for assessing disease ecology and informing livestock health management strategies.

Conducting epidemiological surveys in remote settings can be difficult due to the lack of adequate facilities for sample processing, cold chain maintenance, and pathogen diagnosis. Using dried blood spots (DBSs) on filter paper provides an easy and cost-effective means of overcoming these challenges. DBSs have been widely used for storing and transporting various human, animal, and plant samples for biochemical assays, therapeutic drug monitoring, and the detection of antigens, nucleic acids, and serological markers for infectious disease diagnosis [15]. To improve access to diagnostic tools for disease surveillance, the World Health Organization has advocated for the use of DBSs for the integrated mapping, monitoring, and surveillance of neglected tropical diseases [16,17], including vector-borne diseases such as dengue, chikungunya, and Zika [18].

This study explored the use of DBSs as a practical surveillance tool for detecting antibodies to AKAV, BTV, and BEFV in feral water buffaloes from remote areas of northern Australia.

## 2. Materials and Methods

### 2.1. Study Area and Design

**Figure 1:**
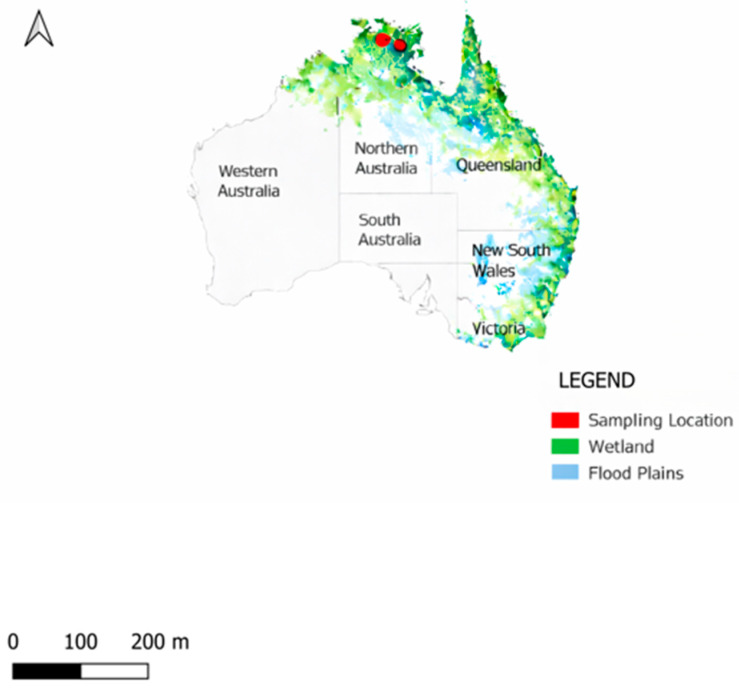
Map of Australia highlighting wetlands and flood plains with clustered sampled areas highlighted in red dots in the Northern Territory. Data source: The Australian Department of Climate Change, Energy, Environment, and Water. https://www.dcceew.gov.au/water/wetlands/publications/directory-important-wetlands, accessed on 1 March 2026.

Convenience sampling was conducted in the Northern Territory, Australia (Figure 1) in August 2023 during the Commonwealth Scientific and Industrial Research Organisation (CSIRO) ‘Space Cows project’ [19] conducted in collaboration with Microsoft^®^. This project aimed to develop satellite-based tracking systems for feral water buffaloes and wild cattle to support Indigenous communities in managing these feral populations for economic, environmental, and cultural benefits [19]. Feral water buffaloes were mustered by helicopter, and Indigenous rangers physically restrained each animal for the attachment of GPS tracking collars. During restraint, blood samples were opportunistically collected by applying blood directly onto Whatman 3MM filter paper (Sigma-Aldrich, St. Louis, MO, USA).

For each sampled buffalo, the following data were recorded: age category (young or adult), determined using a combination of body size, degree of horn development, tooth eruption patterns, tooth wear [10]; sex; body condition score [11]; and GPS coordinates. Dried blood spots (DBSs) were placed in individual sealed plastic bags with desiccant, transported to the Australian Institute of Tropical Health and Medicine laboratory in Townsville, Australia, and stored at −20 °C until serological analysis.

### 2.2. Detection of Arboviral Antibodies in DBSs

Before processing, DBS samples were removed from −20 °C storage and allowed to equilibrate to room temperature for 30 min. A 6 mm Whatman WB10040 Harris Unicore puncher was used to punch two circles from each dried blood spot [12]. The quantity of serum present in each circular segment of filter paper was determined using the equation: 0.5 × the volume of blood per spot divided by the total number of filter paper circles per spot. Consequently, each circular segment of the filter paper contained approximately 10–12 µL of serum (0.5 × [60–70 µL/3]), which is analogous to the requisite volume (10 µL) for serum ELISAs [12]. Between samples, the puncher was cleaned with 70% ethanol-soaked paper towels to prevent cross-contamination.

Each set of two punched discs was placed in a 1.5 mL microcentrifuge tube, and the appropriate ELISA-specific dilution buffer was added according to the manufacturer’s instructions for each assay. Tubes were incubated at room temperature on a Grant-bio PMR-100 Rocker Shaker (Grant Inc, Beaver Falls, PA, USA) for 2 h at 50 revolutions per minute (RPM) and an angle of 10 degrees to elute antibodies from the dried blood matrix. Following incubation, tubes were centrifuged at 10,000× *g* for 5 min using a Beckman Coulter microfuge (Lane Cove, NSW, Australia) to pellet cellular debris, and the supernatant (eluate) was transferred to a new tube for testing.

Eluates were tested using commercial competitive ELISAs to detect IgG antibodies against AKAV (ID Screen^®^ Akabane competition, ID Vet, Grabels, France), BTV (ID Screen^®^ Bluetongue competition, ID Vet, Grabels, France), and BEFV (Bovine Ephemeral Fever Virus Antibody Test Kit, ELISA, AFG Bioscience^®^, IL, USA) following the manufacturers’ protocols. Each ELISA plate included manufacturer-supplied positive and negative control sera, which were reconstituted and handled according to package instructions. Optical density (OD) values were measured at 450 nm using a FLUOstar Omega microplate reader (BMG Labtech Pty Ltd., Mornington, Victoria, Australia).

For the AKAV ELISA, test validity required that the mean OD of the negative control (OD_NC_) was >0.600 and the ratio OD_PC_/OD_NC_ was <0.500 (where OD_PC_ = mean OD of positive control). Sample results were calculated as percentage competition: (ODsample/OD_NC_) × 100. Samples with ≤30% competition were classified as positive, while those with >30% competition were classified as negative.

For the BTV ELISA, test validity required that OD_NC_ was >0.700 and the ratio OD_PC_/OD_NC_ was <0.300. Sample results were calculated as percentage competition: (ODsample/OD_NC_) × 100. Samples with <40% competition were classified as positive, while those with ≥40% competition were classified as negative.

For the BEFV ELISA, test validity required that the mean OD of the positive control was ≥1.000 and the mean OD of the negative control was ≤0.100. The cut-off value was calculated as: mean OD_NC_ + 0.150. Samples with OD values ≥ cut-off were classified as positive, while those with OD values <cut-off were classified as negative.

Plates that failed to meet validity criteria were repeated. All samples were tested in duplicate, and samples with discordant results between duplicates were retested.

### 2.3. Data Analysis

Descriptive statistics were calculated for seroprevalence of each virus, presented as frequencies and percentages with exact binomial (Clopper–Pearson) 95% confidence intervals. Where cell counts were <5, Chi-square tests or Fisher’s exact tests were used to compare seroprevalence between categorical variables, including age category (young vs. adult), sex (male vs. female), and body condition score.

To visualise co-exposure patterns, we identified individual buffaloes with antibodies to multiple viruses and displayed the overlap using a Venn diagram.

True prevalence (Tp) for each virus was estimated from the apparent prevalence (Ap) using the Rogan–Gladen estimator [13], which adjusts for diagnostic test sensitivity (Se) and specificity (Sp):

(1) $$Tp\;=\frac{\left(Ap\;+\;Sp\;-\;1\right)}{\left(Se\;+\;Sp\;-\;1\right)}.$$

Manufacturer-reported test performance characteristics for sera were used for calculations: AKAV ELISA (Se = 1.00, Sp = 1.00), BTV ELISA (Se = 1.00, Sp = 1.00), and BEFV ELISA (Se = 0.93, Sp = 0.95). Confidence intervals for true prevalence (Supplementary Table S1) were calculated using the method described by [20]. All analyses were performed using R version 4.3.3 (R Core Team, Vienna, Austria).

## 3. Results

**Figure 2:**
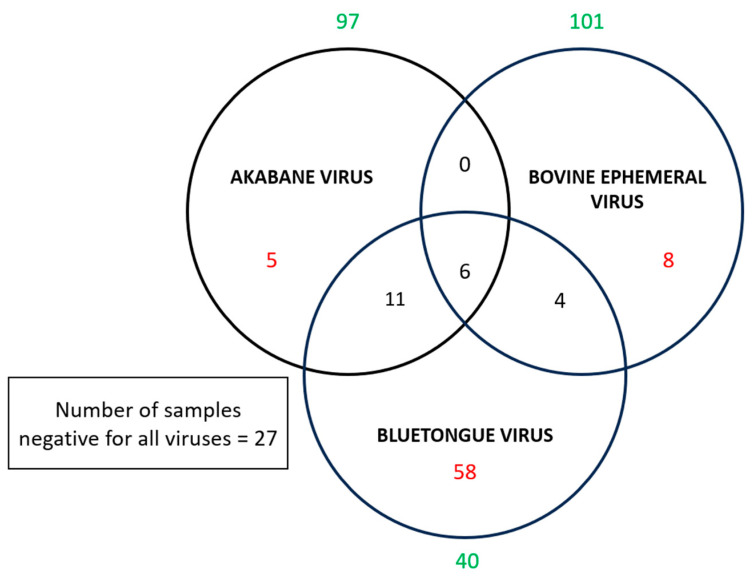
Venn diagram showing co-exposure patterns to Akabane virus (AKAV), bluetongue virus (BTV), and bovine ephemeral fever virus (BEFV) in feral water buffaloes from the Northern Territory, Australia. The numbers in red colour indicate buffaloes with antibodies to single viruses, co-exposures of multiple viruses are indicated in black colour (in intersecting regions), and green colour indicates the buffaloes that tested negative for each virus.

DBS were collected from 119 feral water buffaloes across two locations in the Northern Territory (Figure 2). The sample comprised approximately equal proportions of females and males (60 females, 59 males) and adults and juveniles (68 adults, 51 juveniles). Body condition scores were classified as excellent in 71 (59.7%) animals, good in 40 (33.6%) animals, and medium in 8 (6.7%) animals. Seroprevalence for individual viruses was as follows (Table 1): AKAV 18.5% (22/119, 95% CI: 11.96–26.64), BTV 66.4% (79/119, 95% CI: 57.15–74.78), and BEFV 15.1% (18/119, 95% CI: 9.22–22.85).

Seroprevalence varied by sex, age, and body condition, though no statistically significant associations were observed (Table 1). Among females, 20.0% (12/60) were seropositive for AKAV compared to 16.9% (10/59) of males, while BTV seropositivity was higher in females (73.3%, 44/60) than in males (59.3%, 35/59). BEFV antibodies were detected in 13.3% (8/60) of females and 16.9% (10/59) of males. Adult buffaloes showed higher seroprevalence for AKAV (20.6%, 14/68) and BTV (73.5%, 50/68) compared to juveniles (AKAV: 15.7%, 8/51; BTV: 56.9%, 29/51), while BEFV seroprevalence was similar between age groups (adults: 14.7%, 10/68; juveniles: 15.7%, 8/51). Buffaloes in medium body condition exhibited the highest seroprevalence across all three viruses (AKAV: 37.5%, 3/8; BTV: 75.0%, 6/8; BEFV: 25.0%, 2/8), though the small sample size in this category limits interpretation.

Co-exposure to multiple viruses was common among seropositive buffaloes (Figure 1). Eleven buffaloes (9.2%) had antibodies to both AKAV and BTV, four (3.4%) to both BEFV and BTV, and none (0%) to both BEFV and AKAV. Six buffaloes (5.0%) were seropositive for all three viruses, while 71 (59.7%) buffaloes had antibodies to only one virus, and 27 (22.7%) buffaloes were seronegative for all three viruses tested.

## 4. Discussion

This study provides the first serological evidence of exposure to AKAV, BTV, and BEFV in feral water buffaloes in the Northern Territory, Australia, with sample prevalence estimates of 18.5%, 66.4%, and 15.1%, respectively. While these three arboviruses are well-recognised pathogens of cattle and other domestic ruminants in Australia, surveillance has historically focused on livestock populations, with limited investigation of wildlife reservoirs. Globally, serological studies of arboviruses in water buffaloes remain scarce. Reported BTV seroprevalence in buffalo populations across Africa, Europe, and Asia ranges from 29% to 91% [20,21,22,23], while antibodies to AKAV and BEFV in buffaloes have only been documented in Tanzania over three decades ago, with prevalence estimates of 54% and 19%, respectively [23]. The detection of antibodies to these arboviruses in feral buffaloes suggests they may potentially serve as maintenance hosts during inter-epizootic periods, though their role in virus amplification and transmission to livestock requires further investigation [24].

The epidemiology of AKAV, BTV, and BEFV in Australia is strongly influenced by climatic and environmental factors that vary markedly between geographic regions. Studies in cattle have demonstrated clear seasonal patterns in temperate southern regions, whereas transmission dynamics in tropical northern Australia remain less well characterised, likely due to more stable year-round conditions that are favourable for vector activity [7]. Temperature and rainfall are key drivers of arbovirus circulation, influencing both vector abundance and host-vector contact rates. For example, temperate regions of Australia experience extreme temperature fluctuations (from >40 °C to below freezing), whereas northern tropical regions have relatively stable temperatures but highly variable rainfall, with individual months receiving up to 600 mm or experiencing severe drought [7]. Historical BEFV epizootics between 1936 and 1976 were associated with periods of abnormally high rainfall linked to La Niña phases of the Southern Oscillation Index [24]. Additionally, wind-borne dispersal of infected vectors has facilitated the introduction of BTV and Japanese encephalitis virus into previously unaffected regions [25,26], highlighting the complex interplay between weather patterns, geography, vector distribution, and arboviral transmission. Ecological overlap of livestock, feral buffalo and vector habitats, together with seasonal climatic drivers, determines when and where viruses circulate in northern tropical Australia. Vector abundance and activity are strongly shaped by temperature, rainfall and flooding, with northern Australia showing less pronounced latitudinal seasonality than southern regions with more transmission in wetter, warmer months [27,28]. Northern cattle grazing areas and feral buffalo ranges intersect with habitats favourable to *Culicoides* and mosquito breeding (e.g., coastal wetlands, floodplains and irrigated/pasture areas), creating opportunities for shared exposure and cross-species transmission [29].

*Culicoides brevitarsis* (biting midges) is the primary vector for BTV and AKAV in Australia, though other *Culicoides* species, including *C. fulvus*, *C. actoni*, and *C. wadai*, also contribute to BTV transmission [30,31]. BEFV is transmitted primarily by *Culex annulirostris*, an abundant mosquito species present across more than 80% of the Australian continent [32]. Both *Culicoides* and *Culex* vectors exhibit specific breeding habitat preferences: *C. brevitarsis* breeds in dung-rich sites [33], while *Cx. annulirostris* utilises diverse aquatic habitats including grasslands, ponds, swamps, and wetlands, with populations peaking following heavy rainfall [34]. Feral water buffaloes in northern Australia inhabit tropical savanna ecosystems characterised by monsoon rainforest patches, floodplain grasslands, and mangrove forests. These wetland-associated habitats not only support large buffalo populations but also provide ideal breeding sites for arbovirus vectors, facilitating year-round transmission. The overlap of buffalo habitat with cattle grazing areas in northern Australia raises concerns about potential spillover or shared transmission cycles between feral and domestic ruminants. There was co-infection of the viruses investigated in the sampled water buffaloes represented in the Venn diagram, illustrating that multiple pathogens may circulate during clinical outbreaks and complicate diagnosis and control [35].

This study has several limitations. The convenience sampling approach and relatively small sample size (n = 119) limit the generalizability of our findings and may not fully represent arbovirus exposure across all feral buffalo populations in tropical Australia. Future studies should increase sample size and cover more regions and seasons. Also, we could not include ecological and environmental covariates (habitat types and climate data) in our analysis, which future research should consider. The cross-sectional study design provides a snapshot of seroprevalence but cannot capture temporal dynamics or seasonal transmission patterns. Longitudinal surveillance incorporating both serological and molecular diagnostic methods would enable the detection of active infections, estimation of incidence rates, and characterisation of seasonal fluctuations in virus circulation, although this will require substantial resources to achieve. Furthermore, viral sequencing would provide valuable insights into strain diversity, evolutionary relationships, and potential transmission links between buffalo and livestock populations.

An additional limitation is the lack of validation of DBS performance characteristics for the specific ELISAs compared to standard serum-based testing. While DBS have been validated for numerous infectious disease assays in humans and livestock [36], assay-specific validation is important to confirm diagnostic sensitivity and specificity when using alternative sample matrices. Future studies should compare detection in both DBSs and serum, along with their diagnostic sensitivity and specificity. Despite these limitations, our study demonstrates the practical utility of DBSs as a surveillance tool for arboviral antibody detection in wildlife populations in remote settings. DBS collection is simple, requires minimal equipment and training, eliminates the need for cold chain maintenance during field collection, and facilitates sample transport and storage under challenging logistical conditions. These advantages make DBSs particularly valuable for expanding the surveillance capacity in remote tropical regions of Australia, where traditional venipuncture and serum separation are impractical.

In conclusion, this study provides evidence that feral water buffaloes in northern Australia are exposed to three economically important arboviruses that affect livestock. The high seroprevalence of BTV (66.4%) and moderate prevalence of AKAV and BEFV suggest active circulation of these viruses in buffalo populations inhabiting vector-rich environments. Given the spatial overlap between buffalo and cattle in northern Australia, further research is needed to assess the epidemiological significance of buffalo as potential reservoir or amplifying hosts and their contribution to livestock disease risk. Our findings highlight the value of DBSs as a practical, cost-effective surveillance tool for detecting arboviral exposure in wildlife populations in remote tropical regions, with potential applications for integrated One Health surveillance approaches.

## Figures and Tables

**Table 1:** Apparent seroprevalence of three arboviruses in convenience-sampled feral water buffaloes in Northern Territory, Australia.

Pathogen	Variable	Samples	n, Prevalence (%)	95%CI	*p*-Value	OR	95%CI for OR
AKAV	Overall	119	22 (18.49)	11.96–26.64			
	Sex						
	Female	60	12 (20)	10.78–32.33	Ref		
	Male	59	10 (16.95)	8.44–28.97	0.880	0.93	0.36–2.36
	Age						
	Adult	68	14 (20.58)	11.74–32.12	Ref		
	Young	51	8 (15.68)	7.02–28.59	0.634	0.71	0.28–1.87
	Body condition						
	Excellent	71	9 (12.68)	5.96–22.70	Ref		
	Good	40	10 (25)	12.69–41.19	0.12	2.30	0.84–6.25
	Medium	8	3 (37.5)	8.52–75.51	0.10	4.13	0.94–21.21
BTV	Overall	119	79 (66.39)	57.15–74.78			
	Sex						
	Female	60	44 (73.33)	60.43–83.93	Ref		
	Male	59	35 (59.32)	45.75–71.93	0.12	0.53	0.24–1.14
	Age						
	Adult	68	50 (73.53)	61.43–83.50	Ref		
	Young	51	29 (56.86)	42.25–70.65	0.08	0.47	0.22–1.03
	Body condition						
	Excellent	71	46 (64.79)	52.54–75.76	Ref		
	Good	40	27 (67.50)	50.87–81.43	0.84	1.13	0.50–2.57
	Medium	8	6 (75)	34.91–96.81	0.71	1.63	0.31–8.69
BEFV	Overall	119	18 (15.13)	9.22–22.85			
	Sex						
	Female	60	8 (13.33)	5.94–24.59	Ref		
	Male	59	10 (16.95)	8.44–28.97	0.62	1.33	0.48–3.64
	Age						
	Adult	68	10 (14.71)	7.28–25.39	Ref		
	Young	51	8 (15.69)	7.02–28.59	1.00	1.08	0.39–2.96
	Body condition						
	Excellent	71	13 (18.31)	10.13–29.27	Ref		
	Good	40	3 (7.5)	1.57–20.39	0.16	0.36	0.10–1.36
	Medium	8	2 (25)	3.19–65.09	0.64	1.49	0.27–8.22

CI = Confidence interval; OR = odds ratio.

## Data Availability

The original contributions presented in this study are included in the article/Supplementary Material. Further inquiries can be directed to the corresponding author(s).

## Supplementary Materials

The following supporting information can be downloaded at: https://www.mdpi.com/article/10.3390/v18030363/s1. Table S1: True prevalence of arboviruses amongst feral water buffalo in Northern Tropical Australia based on the manufacturer’s diagnostic sensitivity (Dse) and specificity (Dsp) for serum.
