# Supplementary material for: Serological Evidence of Akabane, Bluetongue, and Bovine Ephemeral Fever Virus Exposure in Feral Water Buffaloes from Northern Australia
Source: Viruses. 2026 Mar 16;18(3):363. doi: 10.3390/v18030363 (PMC13030519; doi:10.3390/v18030363)
Supplement: Supplementary file 1 [file viruses-18-00363-s001.zip › viruses-4068493-supplementary.pdf]

Table S1: True prevalence of arboviruses amongst feral water buffalo in Northern Tropical Australia based on the manufacturer's diagnostic sensitivity (Dse) and specificity (Dsp) for serum

Pathogen	True prevalence (%)	95% CI for TP
Akabane virus	15.33	7.91-24.59
Bluetongue virus	66.39	57.15 - 74.78
Bovine ephemeral fever virus	15.13	9.22 - 22.85
